# Recent Developments in the Metal-Catalyzed Synthesis of Nitrogenous Heterocyclic Compounds

**DOI:** 10.3390/molecules29225458

**Published:** 2024-11-19

**Authors:** Xueguo Zhang, Wenxuan Bi, Zhenyu Cao, Jian Shen, Baohua Chen

**Affiliations:** 1 Department of Materials Science and Engineering, Liaocheng University, Liaocheng 252000, China; 2 Shandong Juxin New Materials Co., Ltd., Zibo 255000, China; 3 State Key Laboratory of Applied Organic Chemistry, Lanzhou University, Lanzhou 730000, China

**Keywords:** metal catalyzed, cyclization, C–H activation, *N*-heterocycles, organic synthesis

## Abstract

Metal-catalyzed cyclization reactions have become a powerful and efficient approach for the stereoselective construction of both carbocyclic and heterocyclic ring systems. Transition metal complexes, with their ability to activate and selectively functionalize organic substrates, have revolutionized various areas of synthetic chemistry. This review highlights recent advancements in metal-catalyzed cyclization reactions, especially in the synthesis of nitrogen-containing heterocycles like imidazoles, pyridines, pyrimidines, and indoles. These advancements have significantly impacted fields such as natural product synthesis, pharmaceuticals, functional materials, and organic electronics. Novel catalytic systems, ligand designs, and reaction conditions continue to expand the capabilities of these reactions, driving further the progress made in synthetic organic chemistry. This review provides a comprehensive overview of recent research.

## 1. Introduction

**Scheme 1:**
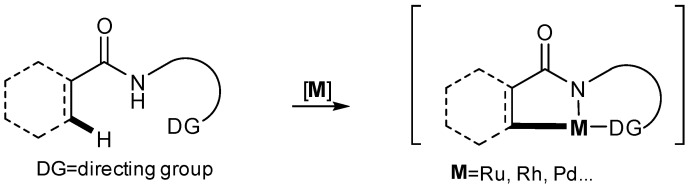
Transition-metals catalyze selective hydrocarbon activation.

The metal-catalyzed cyclization reaction has a long and rich history in organic synthesis, with its importance increasing significantly over time. This approach has become an efficient and powerful method for the stereoselective construction of both carbocyclic and heterocyclic ring systems [1,2,3,4]. Transition metal complexes can activate and selectively functionalize organic substrates, revolutionizing many areas of synthetic chemistry. These catalysts facilitate a wide range of cyclization reactions, enabling the rapid assembly of complex cyclic structures that are challenging to construct through conventional methods (Scheme 1) [5,6,7,8]. By precisely controlling the coordination and reactivity of the metal center, chemists can guide the cyclization reaction to achieve the desired stereochemical outcome, leading to the formation of target cyclic compounds. The versatility and utility of transition metal catalysts have made them indispensable tools for modern organic chemists [9,10,11].

**Scheme 2:**
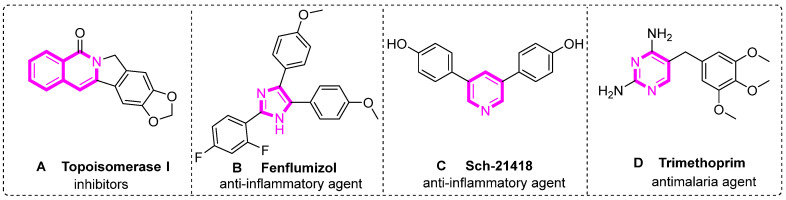
Representative nitrogen-containing heterocyclic pharmaceutical molecules.

The applications of transition metal-catalyzed cyclization reactions span diverse fields, from the synthesis of natural products and pharmaceuticals to the preparation of functional materials and organic electronics. For instance, these processes have been indispensable in synthesizing drug molecules **A**–**D** (Scheme 2) [12,13,14,15]. The efficiency and selectivity of these transformations make them invaluable in the total synthesis of complex natural products, where constructing intricate ring systems is often crucial [16,17,18,19,20]. Additionally, the ability to fine-tune the catalytic system has led to increasingly selective and chemoselective cyclization reactions, allowing for the formation of specific cyclic scaffolds even in the presence of multiple reactive functional groups. Looking to the future, the advancement of transition metal catalysis in organic synthesis promises to unlock new levels of molecular complexity and diversity [21,22,23]. As researchers explore novel catalytic systems, ligand designs, and reaction conditions, the scope and capabilities of metal-catalyzed cyclization reactions will continue to expand, driving further the progress made in synthetic organic chemistry.

In recent years, our research group has focused on exploring the formation of nitrogen-containing heterocycles, such as imidazoles, pyridines, pyrimidines, and indoles, through metal-catalyzed methods. This discussion will primarily examine synthetic methods for these compounds and highlight the related work conducted by our team.

## 2. Transition Metal-Catalyzed Synthesis of Nitrogen-Containing Heterocyclic Compounds

**Scheme 3:**
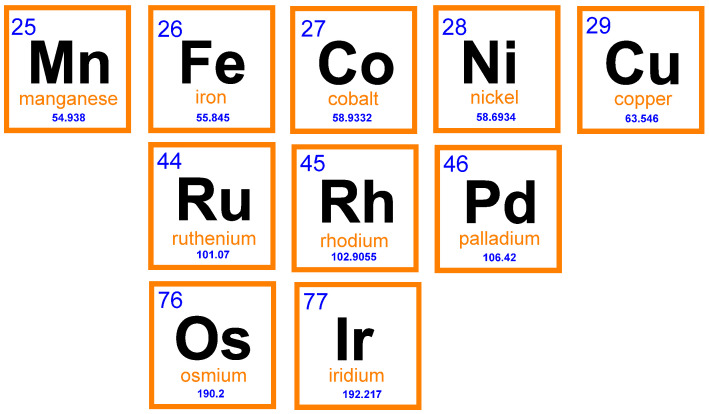
Commonly used transition metals.

Transition metals play a pivotal role in organic synthesis due to their unique electronic properties and ability to facilitate a wide range of chemical transformations (Scheme 3) [24]. These metals serve as catalysts in various reactions, enhancing reaction rates and selectivity.

### 2.1. Manganese (Mn)-Catalyzed Cyclization

**Scheme 4:**
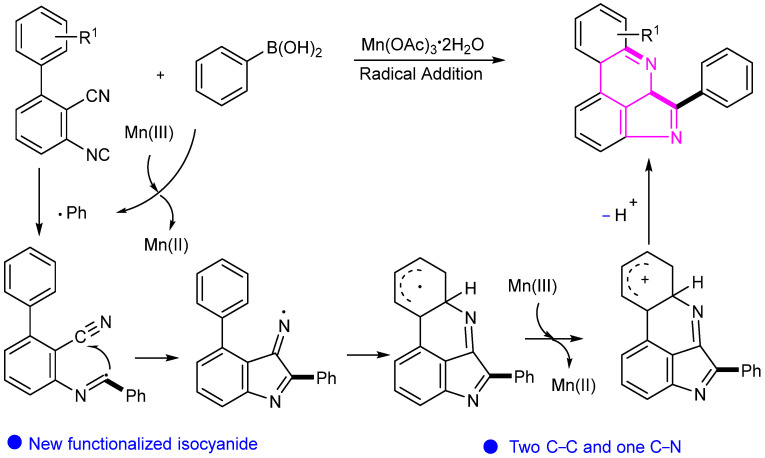
Proposed mechanism for the synthesis of pyrrolopyridine derivatives.

In 2019, Ji’s group reported a Mn-catalyzed cyclization method for synthesizing pyrrolopyridine derivatives from 3-isocyano-[1,1′-biphenyl]-2-carbonitrile and arylboronic acid (Scheme 4) [25]. A series of pyrrolopyridine compounds were constructed through the formation of two new C–C bonds and one C–N bond via a radical pathway. The proposed reaction mechanism clearly illustrates the entire conversion process.

**Scheme 5:**
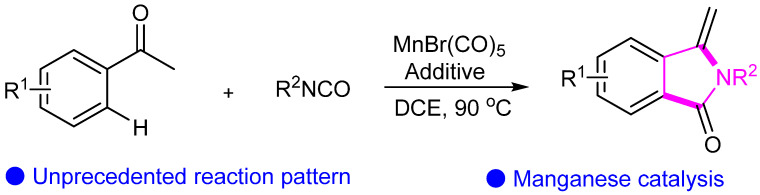
Manganese-catalyzed cyclization of ketones and isocyanates.

In 2021, Wang’s group presented Mn-catalyzed [3 + 2] cyclization reaction involving ketones and isocyanates through inert C–H activation (Scheme 5) [26]. The method not only offers an efficient synthetic route but also underscores the catalytic role of manganese in this transformation. Through detailed experimental validation and exploration of the reaction mechanism, the distinctive features and potential applications of this reaction are elucidated. This research introduces a new paradigm in the field of manganese-catalyzed oxidative cyclization reactions, offering valuable insights for further research and development in related areas.

### 2.2. Iron (Fe)-Catalyzed Cyclization

**Scheme 6:**
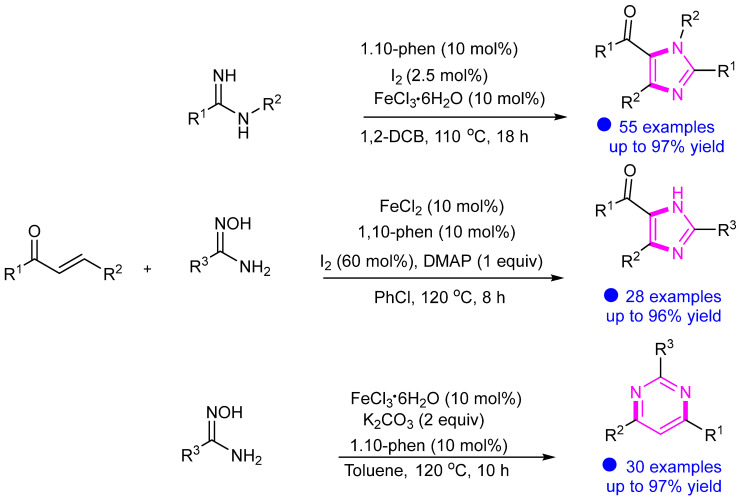
Synthesis of imidazole and pyrimidine compounds catalyzed by iron.

As an inexpensive metal, iron catalysis plays an important role in the synthesis of many nitrogen-containing heterocyclic compounds. In 2015 and 2018, we reported iron-catalyzed syntheses of highly substituted imidazoles and pyrimidines from readily available *α,β*-unsaturated ketones and amidines (Scheme 6) [27,28]. In these reactions, two C–N bonds were simultaneously constructed via metal catalysis.

**Scheme 7:**
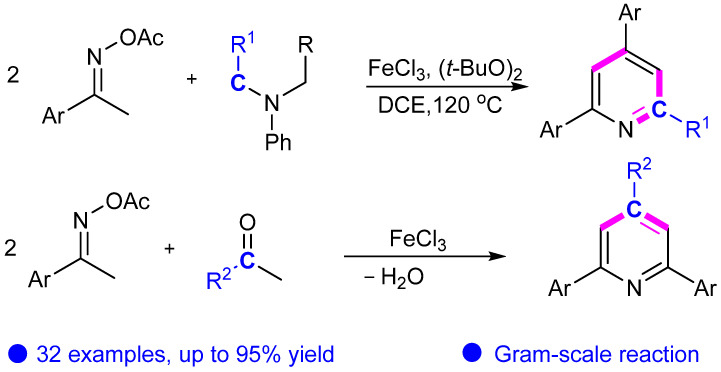
Synthesis of pyridine compounds catalyzed by iron.

In 2016, Guan’s group developed a novel and efficient iron-catalyzed cyclization of ketoxime carboxylates with dialkylanilines or aldehydes (Scheme 7) [29,30]. The reaction begins with the cleavage of the N–O bond in ketoxime carboxylates, facilitated by an iron catalyst. The catalytic effect of iron is highly effective, resulting in a reaction yield of up to 95%. In addition, the product yield of the gram-scale reaction is also excellent.

**Scheme 8:**
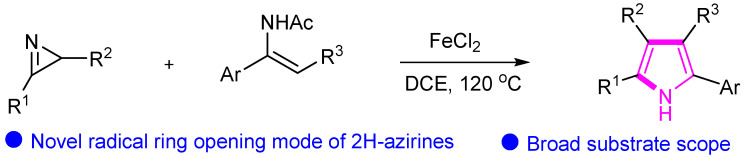
Synthesis of pyrrole compounds catalyzed by iron.

In 2018, Guan’s group also described the iron-catalyzed radical cycloaddition of 2*H*-azirines and enamides as a novel strategy for the synthesis of pyrroles (Scheme 8) [31]. The efficient protocol showcases the versatility of iron catalysis in the construction of pyrrole frameworks. Through a systematic investigation of the reaction conditions and mechanistic pathways, this research elucidates the synthetic route and highlights the significance of iron catalysis in enabling the formation of pyrroles. The developed methodology offers a valuable tool for accessing diverse pyrrole derivatives and contributes to the advancement of synthetic methodologies in heterocyclic compound synthesis.

### 2.3. Cobalt (Co)-Catalyzed Cyclization

**Scheme 9:**
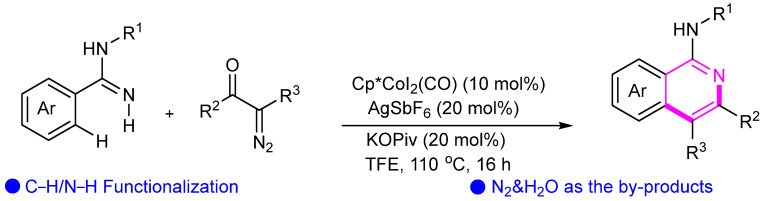
Cobalt(III)-catalyzed synthesis of isoquinolines through C–H functionalization.

Cobalt catalysts are commonly used in C–H activation to target specific hydrogen sites. Utilizing a direct C–H activation strategy, an efficient Co-catalyzed redox-neutral [4 + 2] annulation of aryl amidines and diazo compounds has been accomplished (Scheme 9) [32]. The reaction proceeds under mild conditions, eliminates the need for oxidants, produces only N_2_ and H_2_O as byproducts, and features a broad substrate scope.

**Scheme 10:**
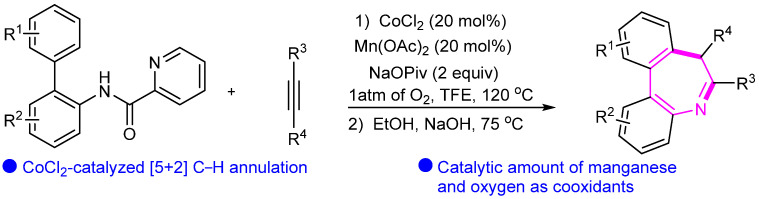
Cobalt(III)-catalyzed synthesis of dibenzo-[b,d]azepines.

Zhong’s group also established Co(II)-catalyzed regioselective and stereoselective [5 + 2] C(*sp*^2^)–H annulation of *o*-arylanilines with alkynes through sequential C–C/C–N bond formation (Scheme 10) [33].

**Scheme 11:**
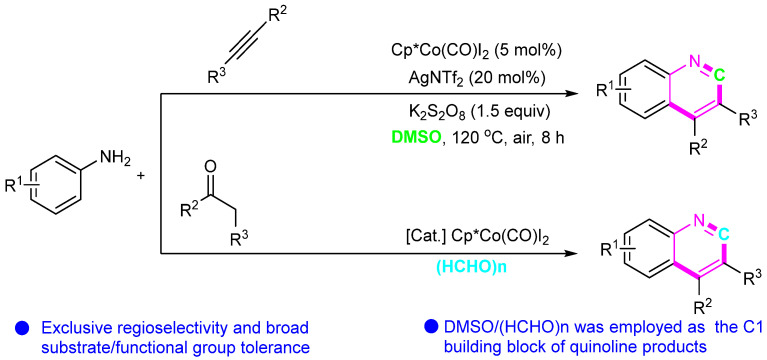
Co(III)-catalyzed and DMSO involved in synthesizing quinolines.

In 2018, Yi’s group developed a protocol for Co-catalyzed annulation reactions between anilines and alkynes involving C–H activation [34,35]. This method features a broad substrate range, a simple operational protocol, and moderate-to-good yields. DMSO/paraformaldehyde liquor served both as the solvent and as the source of the quinoline C1 product (Scheme 11). This method enriches the synthesis methods of quinolines and lays a foundation for their future application.

**Scheme 12:**
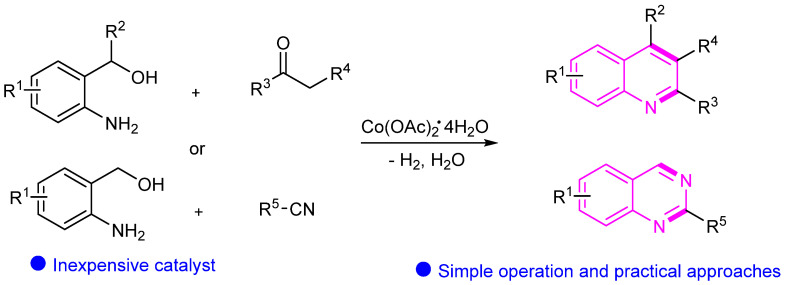
Cobalt-catalyzed synthesis of quinolines and quinazolines.

In 2022, Lu’s group presented cobalt-catalyzed cyclization of 2-aminoaryl alcohols with ketones or nitriles synthesis of quinolines and quinazolines (Scheme 12) [36]. The innovative protocol demonstrates the effectiveness of cobalt catalysis in the construction of these heterocyclic structures. The developed methodology provides a valuable approach for accessing diverse derivatives of quinoline and quinazoline, contributing to the advancement of synthetic strategies in heterocyclic compound synthesis.

### 2.4. Nickel (Ni)-Catalyzed Cyclization

Nickel (Ni) has gained prominence as an alternative to palladium, particularly in recent years. Nickel’s ability to undergo multiple oxidation states makes it suitable for various transformations, including C–H activation and polymerization processes. Its catalytic efficiency in forming complex molecules from simpler substrates is increasingly recognized, making it a valuable tool for synthetic chemists.

**Scheme 13:**
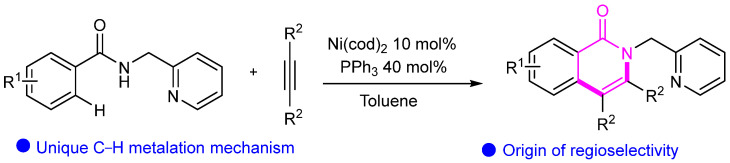
Ni-catalyzed ortho C(*sp*^2^) –H oxidative cycloaddition of aromatic amides.

**Scheme 14:**
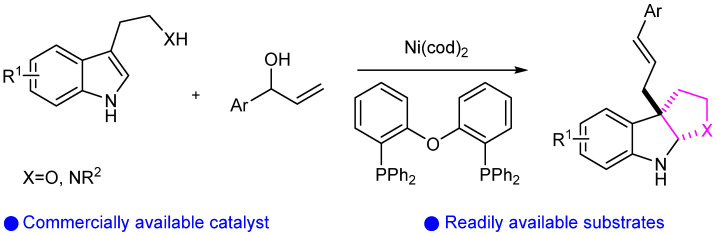
Ni-catalyzed intermolecular allylic dearomatization reaction.

Then, Liu’s group reported the Ni(II)-catalyzed *ortho* C(*sp*^2^)–H oxidative cycloaddition of aromatic amides with alkynes, resulting in the isoquinolone cycloaddition product shown in Scheme 13 [37]. Furthermore, the reaction mechanism was thoroughly studied, promoting the development of other transition metal-catalyzed C–H functionalization reactions with alkynes. Similarly, You’s group reported an efficient method for synthesizing pyrrolidinoindolines via Ni catalysis (Scheme 14) [38].

**Scheme 15:**
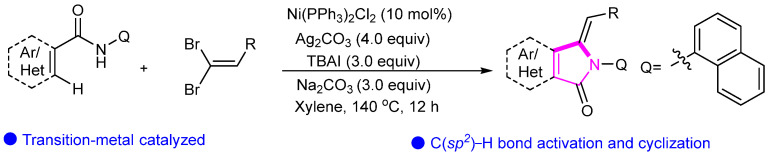
Ni-catalyzed C(*sp*^2^)–H activation and intramolecular annulation.

Additionally, our group disclosed an efficient, operationally simple and scalable nickel-catalyzed inactivated C(*sp*^2^)–H bond activation and cyclization for the synthesis of divergent isoindolinones with excellent selectivity from readily available gem-dibromoalkenes (Scheme 15) [39].

### 2.5. Copper (Cu)-Catalyzed Cyclization

**Scheme 16:**
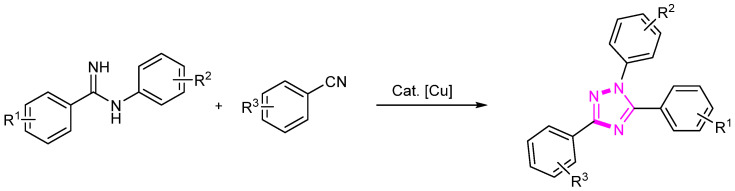
Cu-catalyzed synthesis of triazole compounds.

Copper is another transition metal that has found extensive application in organic synthesis, particularly in the formation of carbon–nitrogen bonds. In 2016, we reported copper-catalyzed synthesis of 1,3,5-trisubstituted 1*H*-1,2,4-triazoles (Scheme 16) [40]. This protocol demonstrates the efficiency of copper catalysis in constructing these valuable heterocyclic compounds. The established methodology provides a direct route for synthesizing diverse derivatives of triazoles, contributing significantly to the advancement of synthetic methodologies in heterocyclic compound synthesis.

**Scheme 17:**
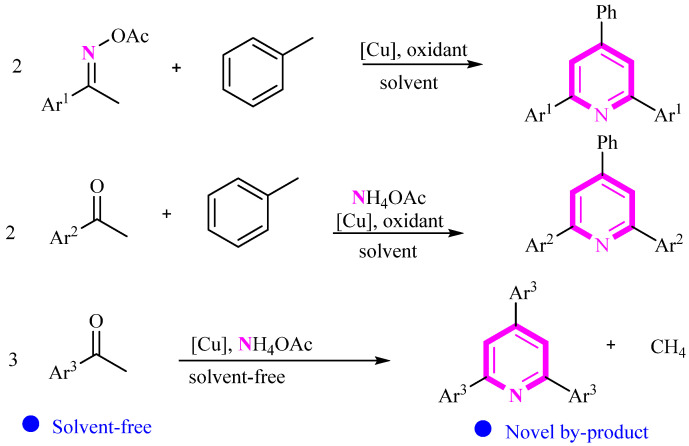
Cu-catalyzed synthesis of polysubstituted pyridine.

Using acetophenone as the raw material, we developed various methods for synthesizing pyridine compounds using copper catalysis (Scheme 17) [41,42,43]. This method is simple to operate and exhibits excellent regioselectivity. Notably, while the third synthesis method is environmentally friendly under solvent-free conditions, it produces a single substituent on the product, limiting diversity compared to the first two methods. Additionally, methane gas is generated in this synthesis, which is a highlight of the paper.

**Scheme 18:**
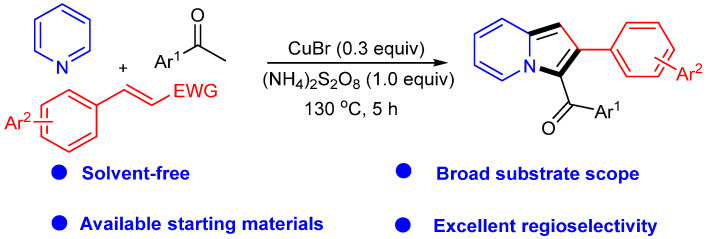
Synthesis of indolizines from pyridine, acetophenone, and electron-deficient alkenes.

Recently, we synthesized indolizine using pyridine, acetophenone, and nitroolefin under CuBr catalysis (Scheme 18) [44]. This reaction underscores the importance of catalysts and oxidants in facilitating chemical transformations and producing valuable organic compounds. Furthermore, the reaction occurs under solvent-free conditions, reflecting environmental friendliness.

### 2.6. Ruthenium (Ru)-Catalyzed Cyclization

The use of inexpensive metals in coupling reactions is widespread, yet noble metals remain essential in many C–H activation reactions due to their excellent coordination ability. Noble metals play an irreplaceable role in catalytic cyclization reactions. Moreover, metal-catalyzed C–H activation/cyclization serves as an attractive alternative to classical cross-coupling reactions, which often require organohalides and organometallic reagents, thanks to the abundance and relatively low cost of various hydrocarbons.

**Scheme 19:**
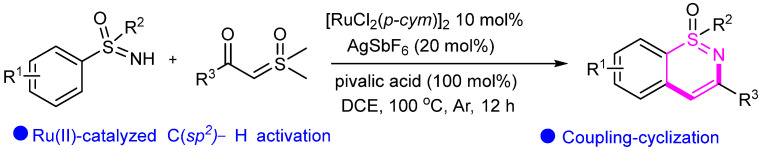
Ru (II)-catalyzed coupling–cyclization reaction.

In 2018, Zeng’s group achieved the synthesis of 1,2-benzothiazines through Ru-catalyzed cyclization of S-phenyl sulfoximine with α-benzoyl sulfoxonium ylide (Scheme 19) [45]. Based on literature reports, the resulting 1,2-benzothiazines can be converted into various 4-substituted analogs for versatile synthetic applications.

**Scheme 20:**
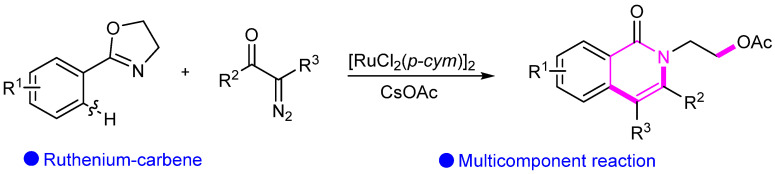
Ruthenium-catalyzed synthesis of isoquinolinones.

Similarly, a Ru(II)-catalyzed approach was developed for the rapid assembly of isoquinolinones, enabling the coupling–cyclization of aryl C(*sp*^2^)–H bonds with diazo compounds via the C(*sp*^2^)–H activation process (Scheme 20) [46].

**Scheme 21:**
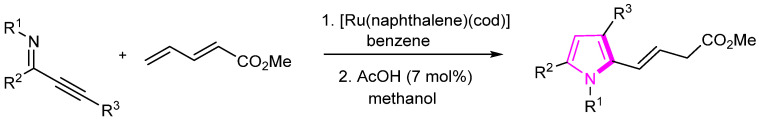
Ru-catalyzed synthesis of pyrrole compounds.

In 2022, Hirano’s group reported Ru-catalyzed synthesis of conjugated iminotrienes followed by an intramolecular cyclization process, leading to the formation of polysubstituted pyrroles (Scheme 21) [47]. The efficient protocol demonstrates the utility of ruthenium catalysis in the construction of complex pyrrole structures. The developed methodology provides a valuable approach for accessing diverse pyrrole derivatives and contributes to the advancement of synthetic strategies in heterocyclic compound synthesis.

### 2.7. Rhodium (Rh)-Catalyzed Cyclization

Rhodium’s ability to activate C–H bonds has opened new avenues for the functionalization of complex organic molecules, further enhancing its significance in organic synthesis. In recent years, the activation and cyclization of hydrocarbons has become a hot research topic in organic synthesis, with numerous results emerging. Our research group has also conducted in-depth studies in this area, and our findings are as follows.

**Scheme 22:**
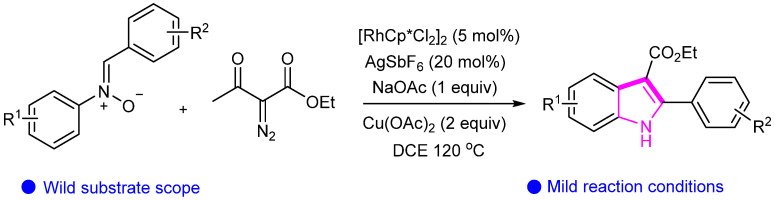
Rhodium(III)-catalyzed C–H activation of arylnitrones and diazo compounds.

We developed a novel strategy for synthesizing indole using arylnitrones and diazo compounds under rhodium catalysis. Compared to traditional coupling reactions, this method is both innovative and practical, providing new insights for activating hydrocarbons to form indole rings. Moreover, a range of functional groups can be applied to this reaction, yielding corresponding products with high selectivity (Scheme 22) [48].

**Scheme 23:**
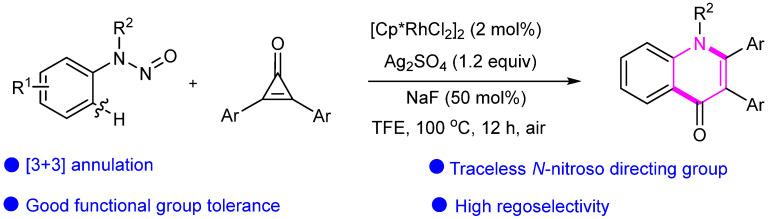
Rh-catalyzed [3 + 3] annulation reactions of *N*-nitrosoanilines and cyclopropenones to obtain functionalized 4-quinolones.

Subsequently, we achieved [3 + 3] cyclization to functionalized 4-quinolones through C(*sp*^2^)–H activation under rhodium catalysis (Scheme 23). This transformation involves the breaking of C–C bonds and the formation of new C–N and C–C bonds, resulting in a target product with good regioselectivity using unsymmetrical meta-substituted *N*-nitrosoanilines. We obtained a variety of quinolinone series compounds with high yields through substrate expansion [49].

**Scheme 24:**
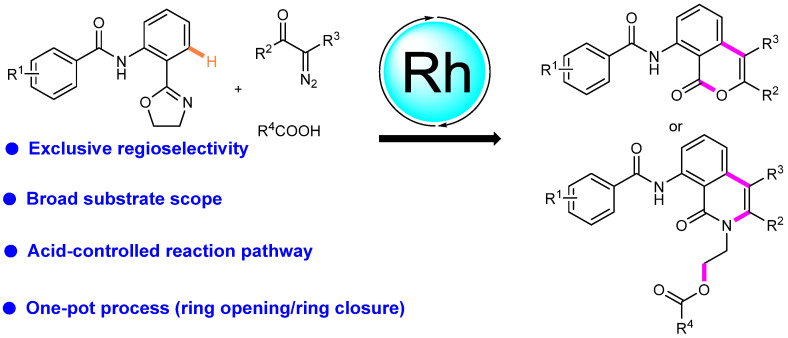
Rhodium(III)-catalyzed annulations between phenyloxazoles and diazo compounds.

In 2021, we successfully devised a straightforward and efficient method for synthesizing isocoumarins and isoquinolinones through a one-pot reaction involving the ring opening and ring closure of phenyloxazoles using diazo compounds (Scheme 24) [50]. This innovative approach, catalyzed by Rh(III), offers the advantage of chemodivergent annulation, allowing for controlled reaction outcomes by adjusting acidity. Moreover, this transformation demonstrates operational simplicity and remarkable compatibility with various functional groups.

**Scheme 25:**
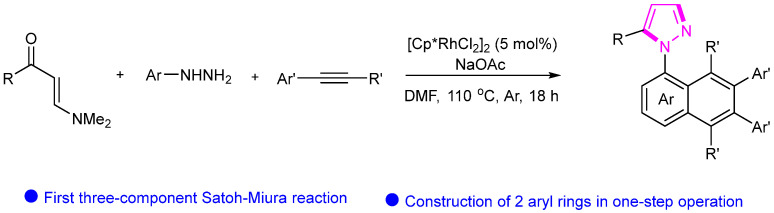
Rh-catalyzed synthesis of pyrazole compounds.

In 2023, Wan’s group reported the three-component synthesis of *N*-naphthyl pyrazoles utilizing a Rh(III)-catalyzed cascade pyrazole annulation and Satoh–Miura benzannulation strategy (Scheme 25) [51]. The innovative protocol showcases the efficacy of Rh(III) catalysis in the construction of these complex heterocyclic compounds. Through a systematic examination of the reaction conditions and mechanistic pathways, this research elucidates the synthetic route and emphasizes the crucial role of Rh(III) catalysis in enabling the formation of N-naphthyl pyrazoles. The developed methodology offers a valuable tool for accessing diverse derivatives of pyrazoles, contributing significantly to the advancement of synthetic methodologies in heterocyclic compound synthesis.

### 2.8. Palladium (Pd)-Catalyzed Cyclization

Palladium is arguably the most prominent transition metal in organic synthesis, particularly in cross-coupling reactions. The Suzuki, Heck, and Sonogashira reactions utilize palladium catalysts to form carbon–carbon bonds efficiently. These reactions are essential for constructing complex molecules, making palladium invaluable in pharmaceutical and materials chemistry. The versatility of palladium also extends to facilitating carbon–heteroatom bond formations. The ability of palladium to adopt various oxidation states and form stable complexes enhances its utility in diverse synthetic pathways.

**Scheme 26:**
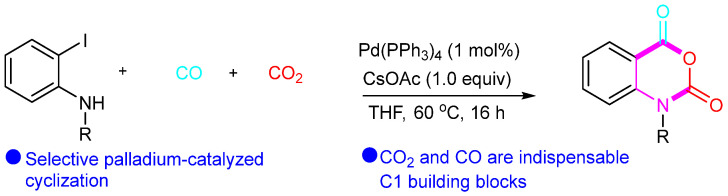
Pd-catalyzed cyclization of *o*-iodoanilines with CO and CO_2_.

**Scheme 27:**
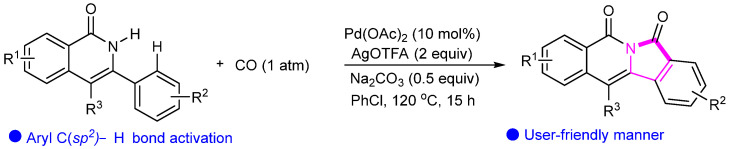
Pd-catalyzed oxidative cyclocarbonylation of isoquinolones with CO.

Lu’s group reported a Pd-catalyzed cyclization reaction for synthesizing isatoic anhydrides from CO_2_, CO, and *o*-iodoanilines (Scheme 26) [52], where CO_2_ and CO served as C1 building blocks for the isatoic anhydride products. In the following year, Fan’s group also reported the synthesis of isoindolo[2,1-b]isoquinoline-5,7-diones through Pd-catalyzed cyclization of isoquinolones with CO (Scheme 27) [53].

**Scheme 28:**
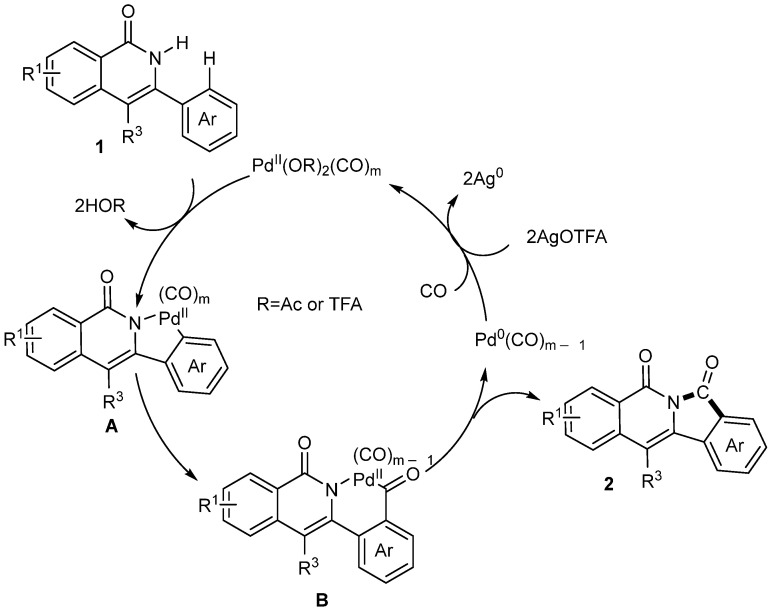
Plausible catalytic cycle.

A mechanism has been proposed for this reaction, as shown in Scheme 25. Firstly, the amide group of **1** chelates with CO-ligated Pd species to give a five-membered palladacycle **A**. Then, the intermediate **A** through migratory insertion affords a six-membered Pd complex **B**. Finally, the intermediate **B** undergoes a reductive elimination to produce product **2** (Scheme 28).

**Scheme 29:**
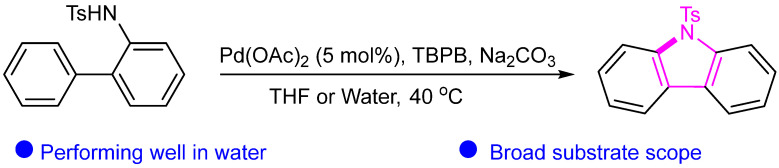
Pd-catalyzed synthesis of carbazole compounds.

In 2022, Xu’s group disclosed the palladium-catalyzed synthesis of carbazoles using peresters as key reactants. The efficient protocol showcases the versatility of palladium catalysis in constructing complex carbazole structures (Scheme 29) [54]. Through a systematic investigation of reaction conditions and mechanisms, this research elucidates the synthetic pathway and highlights the significance of palladium catalysis in carbazole synthesis. The developed methodology provides a valuable tool for accessing carbazole derivatives and contributes to the advancement of synthetic methodologies in heterocyclic compound synthesis.

More importantly, palladium catalysts have shown significant effects in C(sp^3^)–H activation processes. In this regard, Yu’s research group has accomplished noteworthy work, achieving various hydrocarbon functionalization.

**Scheme 30:**
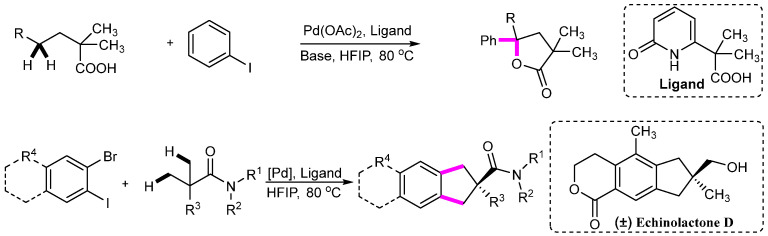
Pd-catalyzed C(*sp*^3^)–H activation.

In 2023, Yu’s group achieved double γ-C(*sp*^3^)–H activation of linear free carboxylic acids under palladium catalysis, along with intramolecular cyclization (Scheme 30) [55]. The coordination of amido-type ligands was key to the success of this reaction, stabilizing the reactive intermediate. In the same year, Yu’s team reported [3 + 2] cyclization involving double *β*-C(*sp*^3^)–H activation under palladium catalysis, representing a crucial step in synthesizing the natural product (±)Echinolactone D (Scheme 30) [56].

### 2.9. Osmium (Os)- and Iridium (Ir)-Catalyzed Cyclization

Osmium and iridium catalysts have become increasingly important in organic synthesis, particularly in cyclization reactions. These precious metal catalysts offer unique reactivity and selectivity that are often irreplaceable in synthesizing complex, specialized compounds. For example, the high oxophilicity of osmium allows it to activate carbonyl groups and other polar functional groups, enabling the formation of new carbon–carbon bonds in a selective and efficient manner. Similarly, iridium catalysts have shown great promise in enantioselective cyclization reactions. The unique electronic and steric properties of iridium can be tailored to achieve highly stereoselective transformations, leading to the formation of complex cyclic structures with precise control over stereochemistry. This capability is particularly valuable in the synthesis of natural products and other biologically active compounds, where the stereochemistry of the final product is often critical to its function [57,58].

**Scheme 31:**
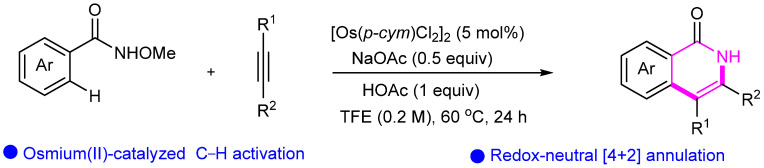
Os-catalyzed synthesis of isoquinolones.

**Scheme 32:**
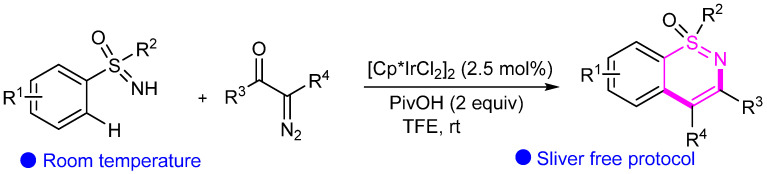
Ir-catalyzed C–H/N–H functionalization of sulfoximines for the synthesis of 1,2-benzothiazines.

Yi’s group developed Os(II)-catalyzed redox-neutral C–H activation/[4 + 2] annulation of N-methoxybenzamides with alkynes for the one-pot assembly of isoquinolones (Scheme 31) [59]. In 2018, Pawar’s group first developed Ir(III)-catalyzed cyclization of sulfoximines with diazocarbonyl compounds for synthesizing 1,2-benzothiazines under redox-neutral conditions (Scheme 32) [60]. This reaction is performed at room temperature and exhibits good yields and functional group tolerance.

## 3. Conclusions

This review highlights the significant role of transition metal-catalyzed cyclization reactions in synthesizing nitrogen-containing heterocycles. These reactions have emerged as powerful tools for the efficient and stereoselective construction of diverse cyclic structures, contributing to various fields, including drug discovery, natural product synthesis, and materials science. The versatility of transition metal catalysts has been demonstrated through numerous examples. Notably, the development of new catalytic systems, ligand designs, and reaction conditions continues to expand the scope and capabilities of metal-catalyzed cyclization reactions, opening new avenues for molecular complexity and diversity. Our group has successfully developed novel methodologies for synthesizing various heterocyclic compounds. These studies demonstrate the potential of both metal-catalyzed and non-metal-catalyzed approaches for efficiently and selectively constructing these valuable building blocks.

Looking forward, continued exploration of both metal-catalyzed and reactions will undoubtedly lead to further advancements in organic synthesis. The development of novel catalysts, reaction conditions, and methodologies will enable the synthesis of a wider range of complex and functionalized heterocycles, paving the way for discovering new drugs, materials, and technologies. At the same time, the ongoing exploration of transition metals will undoubtedly lead to innovative strategies for the synthesis of pharmaceuticals, agrochemicals, and advanced materials, reinforcing their integral role in modern chemistry.

## Data Availability

No new data were created or analyzed in this study.

## References

[1] Neto J.S.S., Zeni G. (2020). Transition Metal-Catalyzed and Metal-Free Cyclization Reactions of Alkynes with Nitrogen-Containing Substrates: Synthesis of Pyrrole Derivatives. ChemCatChem.

[2] Li J., Lin Z., Wu W., Jiang H. (2020). Recent advances in metal catalyzed or mediated cyclization/functionalization of alkynes to construct isoxazoles. Org. Chem. Front..

[3] Lv Y., Meng J., Li C., Wang X., Ye Y., Sun K. (2021). Update on the Synthesis of N-Heterocycles via Cyclization of Hydrazones (2017–2021). Adv. Synth. Catal..

[4] Xu B., Wang Q., Fang C., Zhang Z.-M., Zhang J. (2024). Recent advances in Pd-catalyzed asymmetric cyclization reactions. Chem. Soc. Rev..

[5] Docherty J.H., Lister T.M., McArthur G., Findlay M.T., Domingo-Legarda P., Kenyon J., Choudhary S., Larrosa I. (2023). Transition-Metal-Catalyzed C–H Bond Activation for the Formation of C–C Bonds in Complex Molecules. Chem. Rev..

[6] Huang H.-M., Bellotti P., Glorius F. (2020). Transition metal-catalysed allylic functionalization reactions involving radicals. Chem. Soc. Rev..

[7] Jiao K.-J., Xing Y.-K., Yang Q.-L., Qiu H., Mei T.-S. (2020). Site-Selective C–H Functionalization via Synergistic Use of Electrochemistry and Transition Metal Catalysis. Acc. Chem. Res..

[8] Achar T.K., Maiti S., Jana S., Maiti D. (2020). Transition Metal Catalyzed Enantioselective C(sp^2^)–H Bond Functionalization. ACS Catal..

[9] Rogge T., Oliveira J.C.A., Kuniyil R., Hu L., Ackermann L. (2020). Reactivity-Controlling Factors in Carboxylate-Assisted C–H Activation under 4d and 3d Transition Metal Catalysis. ACS Catal..

[10] Gao W.-X., Feng H.-J., Guo B.-B., Lu Y., Jin G.-X. (2020). Coordination-Directed Construction of Molecular Links. Chem. Rev..

[11] Wang L., Zhu C., Xu M., Zhao C., Gu J., Cao L., Zhang X., Sun Z., Wei S., Zhou W. (2021). Boosting Activity and Stability of Metal Single-Atom Catalysts via Regulation of Coordination Number and Local Composition. J. Am. Chem. Soc..

[12] Pommier Y. (2006). Topoisomerase I inhibitors: Camptothecins and beyond. Nat. Rev. Cancer.

[13] Sharghi H., Razavi S.F., Aberi M. (2021). One-Pot Three-Component Synthesis of 2,4,5-Triaryl-1H-imidazoles Using Mn^2+^ Complex of [7-Hydroxy-4-methyl-8-coumarinyl] Glycine as a Heterogeneous Catalyst. Catal. Lett..

[14] Sathish M., Chetna J., Hari Krishna N., Shankaraiah N., Alarifi A., Kamal A. (2016). Iron-Mediated One-Pot Synthesis of 3,5-Diarylpyridines from β-Nitrostyrenes. J. Org. Chem..

[15] Mahmood Aljamali N., Alwan Alsabri I.K. (2020). Development of Trimethoprim Drug and Innovation of Sulfazane-Trimethoprim Derivatives as Anticancer Agents. Biomed. Pharmacol. J..

[16] Trost B.M., Crawley M.L. (2003). Asymmetric Transition-Metal-Catalyzed Allylic Alkylations: Applications in Total Synthesis. Chem. Rev..

[17] Arcadi A., Morlacci V., Palombi L. (2023). Synthesis of Nitrogen-Containing Heterocyclic Scaffolds through Sequential Reactions of Aminoalkynes with Carbonyls. Molecules.

[18] Wang B., Perea M.A., Sarpong R. (2020). Transition Metal-Mediated C−C Single Bond Cleavage: Making the Cut in Total Synthesis. Angew. Chem..

[19] Mohammadkhani L., Heravi M.M. (2020). Applications of Transition-Metal-Catalyzed Asymmetric Allylic Substitution in Total Synthesis of Natural Products: An Update. Chem. Rec..

[20] Sinha S.K., Ghosh P., Jain S., Maiti S., Al-Thabati S.A., Alshehri A.A., Mokhtar M., Maiti D. (2023). Transition-metal catalyzed C–H activation as a means of synthesizing complex natural products. Chem. Soc. Rev..

[21] Cheng W.-M., Shang R. (2020). Transition Metal-Catalyzed Organic Reactions Under Visible Light: Recent Developments and Future Perspectives. ACS Catal..

[22] Malapit C.A., Prater M.B., Cabrera-Pardo J.R., Li M., Pham T.D., McFadden T.P., Blank S., Minteer S.D. (2021). Advances on the Merger of Electrochemistry and Transition Metal Catalysis for Organic Synthesis. Chem. Rev..

[23] Liu B., Yang L., Li P., Wang F., Li X. (2021). Recent advances in transition metal-catalyzed olefinic C–H functionalization. Org. Chem. Front..

[24] Sohail M., Bilal M., Maqbool T., Rasool N., Ammar M., Mahmood S., Malik A., Zubair M., Abbas Ashraf G. (2022). Iron-catalyzed synthesis of N-heterocycles via intermolecular and intramolecular cyclization reactions: A review. Arab. J. Chem..

[25] Xu P., Zhu Y.-M., Wang F., Wang S.-Y., Ji S.-J. (2019). Mn(III)-Mediated Cascade Cyclization of 3-Isocyano-[1,1′-biphenyl]-2-carbonitrile with Arylboronic Acid: Construction of Pyrrolopyridine Derivatives. Org. Lett..

[26] Huo J., Yang Y., Wang C. (2021). Manganese-Catalyzed [3 + 2] Cyclization of Ketones and Isocyanates via Inert C-H Activation. Org. Lett..

[27] Wu P., Qu J., Li Y., Guo X., Tang D., Meng X., Yan R., Chen B. (2015). Iron(III)/Iodine-Catalyzed C(sp^2^)–H Activation of α,β-Unsaturated Aldehydes/Ketones with Amidines: Synthesis of 1,2,4,5-Tetrasubstituted Imidazoles. Adv. Synth. Catal..

[28] Wu P., Zhang X., Chen B. (2019). Direct synthesis of 2,4,5-trisubstituted imidazoles and di/tri-substituted pyrimidines via cycloadditions of α,β-unsaturated ketones/aldehydes and N′-hydroxyl imidamides. Tetrahedron Lett..

[29] Zhao M.-N., Ren Z.-H., Yu L., Wang Y.-Y., Guan Z.-H. (2016). Iron-Catalyzed Cyclization of Ketoxime Carboxylates and Tertiary Anilines for the Synthesis of Pyridines. Org. Lett..

[30] Yi Y., Zhao M.-N., Ren Z.-H., Wang Y.-Y., Guan Z.-H. (2017). Synthesis of symmetrical pyridines by iron-catalyzed cyclization of ketoxime acetates and aldehydes. Green Chem..

[31] Zhao M.N., Ren Z.H., Yang D.S., Guan Z.H. (2018). Iron-Catalyzed Radical Cycloaddition of 2H-Azirines and Enamides for the Synthesis of Pyrroles. Org. Lett..

[32] Li J., Tang M., Zang L., Zhang X., Zhang Z., Ackermann L. (2016). Amidines for Versatile Cobalt(III)-Catalyzed Synthesis of Isoquinolines through C–H Functionalization with Diazo Compounds. Org. Lett..

[33] Ling F., Xie Z., Chen J., Ai C., Shen H., Wang Z., Yi X., Zhong W. (2019). Cobalt(II)-Catalyzed [5 + 2] C−H Annulation of o-Arylanilines with Alkynes: An Expedient Route to Dibenzo-[b,d]azepines. Adv. Synth. Catal..

[34] Xu X., Yang Y., Zhang X., Yi W. (2018). Direct Synthesis of Quinolines via Co(III)-Catalyzed and DMSO-Involved C–H Activation/Cyclization of Anilines with Alkynes. Org. Lett..

[35] Xu X., Yang Y., Chen X., Zhang X., Yi W. (2017). The one-pot synthesis of quinolines via Co(iii)-catalyzed C-H activation/carbonylation/cyclization of anilines. Org. Biomol. Chem..

[36] Hao Z., Zhou X., Ma Z., Zhang C., Han Z., Lin J., Lu G.L. (2022). Dehydrogenative Synthesis of Quinolines and Quinazolines via Ligand-Free Cobalt-Catalyzed Cyclization of 2-Aminoaryl Alcohols with Ketones or Nitriles. J. Org. Chem..

[37] Omer H.M., Liu P. (2019). Computational Study of the Ni-Catalyzed C–H Oxidative Cycloaddition of Aromatic Amides with Alkynes. ACS Omega.

[38] Zhang H.-J., Gu Q., You S.-L. (2019). Ni-Catalyzed Intermolecular Allylic Dearomatization Reaction of Tryptophols and Tryptamines. Org. Lett..

[39] Shi Y., Li M.-S., Zhang F., Chen B. (2018). Nickel(ii)-catalyzed tandem C(sp^2^)–H bond activation and annulation of arenes with gem-dibromoalkenes. RSC Adv..

[40] Chen B., Zhang L., Tang D., Gao J., Wang J., Wu P., Meng X. (2016). Direct Access to 1,3,5-Trisubstituted 1H-1,2,4-Triazoles from N-Phenylbenzamidines via Copper-Catalyzed Diamination of Aryl Nitriles. Synthesis.

[41] Zhang X., Wang P., Yuan X., Qin M., Chen B. (2019). Synthesis of Pyridine Derivatives from Acetophenone and Ammonium Acetate by Releasing CH4. Asian J. Org. Chem..

[42] Fu Y., Wang P., Guo X., Wu P., Meng X., Chen B. (2016). Synthesis of Polyfunctional Pyridines via Copper-Catalyzed Oxidative Coupling Reactions. J. Org. Chem..

[43] Han J., Guo X., Liu Y., Fu Y., Yan R., Chen B. (2017). One-Pot Synthesis of Benzene and Pyridine Derivatives via Copper-Catalyzed Coupling Reactions. Adv. Synth. Catal..

[44] Zhang X., Zhang J., Liu Z., Bi W., Shen J., Li G. (2024). Efficient Solvent-Free Synthesis of Indolizines Using CuBr Catalyst from Pyridine, Acetophenone, and Electron-Deficient Alkenes. Molecules.

[45] Xie H., Lan J., Gui J., Chen F., Jiang H., Zeng W. (2018). Ru (II)-Catalyzed Coupling-Cyclization of Sulfoximines with alpha-Carbonyl Sulfoxonium Ylides as an Approach to 1,2-Benzothiazines. Adv. Synth. Catal..

[46] Kumar G.S., Khot N.P., Kapur M. (2018). Oxazolinyl-Assisted Ru(II)-Catalyzed C−H Functionalization Based on Carbene Migratory Insertion: A One-Pot Three-Component Cascade Cyclization. Adv. Synth. Catal..

[47] Kurita S., Kiyota S., Komine N., Hirano M. (2022). Ru(0)-Catalyzed Synthesis of Conjugated Iminotrienes and Subsequent Intramolecular Cyclization Giving Polysubstituted Pyrroles. Org. Lett..

[48] Guo X., Han J., Liu Y., Qin M., Zhang X., Chen B. (2017). Synthesis of 2,3-Disubstituted NH Indoles via Rhodium(III)-Catalyzed C–H Activation of Arylnitrones and Coupling with Diazo Compounds. J. Org. Chem..

[49] Liu Y., Tian Y., Su K., Wang P., Guo X., Chen B. (2019). Rhodium(iii)-catalyzed [3 + 3] annulation reactions of N-nitrosoanilines and cyclopropenones: An approach to functionalized 4-quinolones. Org. Chem. Front..

[50] Zhang X., Wang P., Zhu L., Chen B. (2021). Rhodium(III)-catalyzed chemodivergent annulations between phenyloxazoles and diazos via C–H activation. Chin. Chem. Lett..

[51] Chen D., Zhou L., Liu Y., Wan J.P. (2023). Three-component synthesis of N-naphthyl pyrazoles via Rh(III)-catalyzed cascade pyrazole annulation and Satoh-Miura benzannulation. Chem. Commun..

[52] Zhang W.-Z., Zhang N., Sun Y.-Q., Ding Y.-W., Lu X.-B. (2017). Palladium-Catalyzed Cyclization Reaction of o-Iodoanilines, CO_2_, and CO: Access to Isatoic Anhydrides. ACS Catal..

[53] Guo S., Wang F., Sun L., Zhang X., Fan X. (2018). Palladium-Catalyzed Oxidative Cyclocarbonylation of Isoquinolones with CO via C−H/N−H Bond Cleavage: Easy Access to Isoindolo[2,1-b]isoquinoline-5,7-dione Derivatives. Adv. Synth. Catal..

[54] He L., Xu Y. (2022). Palladium-Catalyzed Synthesis of Carbazoles by Perester. Adv. Synth. Catal..

[55] Hoque M.E., Yu J.Q. (2023). Ligand-Enabled Double γ-C(sp^3^)−H Functionalization of Aliphatic Acids: One-Step Synthesis of γ-Arylated γ-Lactones. Angew. Chem..

[56] Tomanik M., Yu J.-Q. (2023). Palladium-Catalyzed Stitching of 1,3-C(sp^3^)–H Bonds with Dihaloarenes: Short Synthesis of (±)-Echinolactone D. J. Am. Chem. Soc..

[57] Crochet P., Cadierno V. (2021). Arene-Osmium(II) Complexes in Homogeneous Catalysis. Inorganics.

[58] Yang F., Xie J.-H., Zhou Q.-L. (2023). Highly Efficient Asymmetric Hydrogenation Catalyzed by Iridium Complexes with Tridentate Chiral Spiro Aminophosphine Ligands. Acc. Chem. Res..

[59] Yang J., Wu L., Xu H., Gao H., Zhou Z., Yi W. (2019). Redox-Neutral [4 + 2] Annulation of N-Methoxybenzamides with Alkynes Enabled by an Osmium(II)/HOAc Catalytic System. Org. Lett..

[60] Aher Y.N., Lade D.M., Pawar A.B. (2018). Cp*Ir(iii)-catalyzed C–H/N–H functionalization of sulfoximines for the synthesis of 1,2-benzothiazines at room temperature. Chem. Commun..

